# Hand-Book of Practical Medicine

**Published:** 1886-11

**Authors:** J. L. Gray


HAND-BOOK OF PRACTICAL MEDICINE. *By* DR. HERMAN EICHHORST, *Professor of Special Pathology and Therapeutics and Director of the University Medical Clinic in Zurich. Volume II.* DISEASES OF THE DIGESTIVE, URINARY AND SEXUAL APPARATUS. *One hundred and six wood engravings. New York:* WM. WOOD & COMPANY, 1886. *Chicago:* W. T. KEENER.

In these times of active business push and enterprise, the average individual pays but very little attention to the requirements or the preservation of a sound physical constitution.

Any manual, therefore, which shall make us better acquainted with the pathology and treatment of diseases of the digestive system is to be considered as not only reflecting credit on the author, but as proving a decided benefit to the reader.

The volume, the title of which stands at the head of this notice, is a part of the great Hand-book of Practical Medicine, by Dr. Eichhorst, treating of diseases of the urinary and sexual apparatus and forms the June number of *Wood's Library*.

In the present notice it is desired to direct attention especially to the portion of the book having reference to the digestive apparatus. This consists of the first seven parts of the work.

The first chapter is devoted to diseases of the buccal cavity and salivary glands, the soft palate and the pharynx.

One point that strikes one as being peculiar is the manner in which directions are given regarding the use of medicines, as, for example, on page 16, in the treatment of salivation the author advises the use of atropia sulphate, gr. $^{1}/_{7}$ to f℥ iij water, ½ to one syringeful, subcutaneously.

This method of direction is, in our judgment, to say the least, not wise. It is a well-known fact that hypodermic syringes of this day vary greatly in their calibre, so that what would be a syringeful of one make would barely half fill a syringe of another make.

This indefinite mode of giving directions as to dosage runs through the whole book. As another example of this peculiar method, in the treatment of acute gastritis the author advises the use of salicylate of soda and bicarbonate of soda, equal parts "as much as can be placed on the tip of a knife, every two hours." It may be that in Germany all knives are the same size and the tips of them hold the same quantity of any material, but in this country such directions would hardly be tolerated.

He says, also, that while the bacterium of rheumatic angina has never been discovered, nevertheless, he believes a cold hardly acts in any other way than by favoring the development of bacteria. This statement is a little hard to reconcile with the one a little farther along, in which he says "heredity occasionally plays a part in the etiology."

The chapter on "Dilatation of the Stomach" is especially full concerning its etiology and diagnosis. Coming next to the functional diseases of the stomach, the author gives a due amount of attention to gastralgia. Regarding the diagnosis of this affection, he says "the recognition of gastralgia is not always easy." It must be differentiated from rheumatism of the abdominal muscles; neuralgia of the lower intercostal nerves; circumscribed peritonitis; biliary colic; intestinal colic; with radiated pains.

The limits of the present notice will not permit our following, in detail, the author through the rather lengthy chapters on diseases of the intestines, of the liver and pancreas. The consideration of diseases of the liver is especially full for a manual, such as the one under consideration, though not comparing, of course, with some of the more elaborate treatises on the diseases of that organ.

The portion of the book devoted to diseases of the kidneys is very complete, taken as a whole. The chapter on diseases of the sexual organs is lacking in many essential particulars. But in view of the great abundance of literature on this class of disorders we doubt not the small space given to the subject in this work was intentional. Taken as a whole, we consider this work one of the best which the enterprising proprietors of *Wood's Library* have issued.

J. L. Gray.

